# Supplementary material for: Momentum-resolved fingerprint of Mottness in layer-dimerized Nb3Br8
Source: Nat Commun. 2025 Apr 29;16:4037. doi: 10.1038/s41467-025-58885-1 (PMC12041277; doi:10.1038/s41467-025-58885-1)
Supplement: Supplementary file 1 — Supplementary Information [file 41467_2025_58885_MOESM1_ESM.pdf]

Supplementary Information

Mihir Date,[1,2] Francesco Petocchi,[3] Yun Yen,[4,5] Jonas A. Krieger,[1] Banabir Pal,[1] Vicky Hasse,[6] Emily C. McFarlane,[1] Chris Körner,[7] Jiho Yoon,[1] Matthew D. Watson,[2] Vladimir N. Strocov,[8] Yuanfeng Xu,[9] Ilya Kostanovski,[1] Mazhar N. Ali,[1,10] Sailong Ju,[8] Nicholas C. Plumb,[8] Michael A. Sentef,[11,12] Georg Woltersdorf,[7] Michael Schüler,[4] Philipp Werner,[13] Claudia Felser,[6] Stuart S. P. Parkin,[1] and Niels B. M. Schröter[1,*]

[1] *Max Planck Institut für Mikrostrukturphysik, Weinberg 2, 06120 Halle, Germany*
[2] *Diamond Light Source Ltd, Harwell Science and Innovation Campus, Didcot, OX11 0DE, U.K.*
[3] *Department of Quantum Matter Physics, University of Geneva, 24 Quai Ernest-Ansermet, 1211 Geneva 4, Switzerland*
[4] *Laboratory for Materials Simulations, Paul Scherrer Institute, CH-5232 Villigen PSI, Switzerland*
[5] *École Polytechnique Fédérale de Lausanne (EPFL), CH-1015 Lausanne, Switzerland*
[6] *Max Planck Institute for Chemical Physics of Solids Nöthnitzer Straße 40 01187 Dresden Germany*
[7] *Martin-Luther-Universität Halle-Wittenberg, Von-Danckelmann-Platz 3 06120 Halle (Saale), Germany*
[8] *Swiss Light Source, Paul Scherrer Institute, CH-5232 Villigen PSI, Switzerland*
[9] *Center for Correlated Matter and School of Physics, Zhejiang University, Hangzhou 310058, China*
[10] *Kavli Institute of Nanoscience, Delft University of Technology, Delft 2628 CJ, The Netherlands*
[11] *Institute for Theoretical Physics and Bremen Center for Computational Materials Science, University of Bremen, 28359, Bremen, Germany*
[12] *Max Planck Institute for the Structure and Dynamics of Matter, Center for Free-Electron Laser Science (CFEL), Luruper Chaussee 149, 22761 Hamburg, Germany*
[13] *Department of Physics, University of Fribourg, Fribourg 1700, Switzerland*

Contents

Supplementary Note 1 1

Supplementary Note 2 2

Supplementary Note 3 3

Supplementary Note 4 3

Supplementary Note 5 3

Supplementary Note 6 5

Supplementary Note 7 5

References 6

Supplementary Note 1

The lattice system associated with the trigonal crystal system is either hexagonal (with a prefix 'P' or hP) or rhombohedral (with a prefix 'R' or hR). Therefore, such systems can be represented in hexagonal or rhombohedral coordinates. For a system described by rhombohedral coordinates $u = [a_R, b_R, c_R]^T$, and equivalently by hexagonal coordinates $v = [a_H, b_H, c_H]^T$, the following relation can be used to transform between the two representations.

$$Ou = v \tag{S1a}$$

$$O^{-1}v = u \tag{S1b}$$

*Electronic address: niels.schroeter@mpi-halle.mpg.de

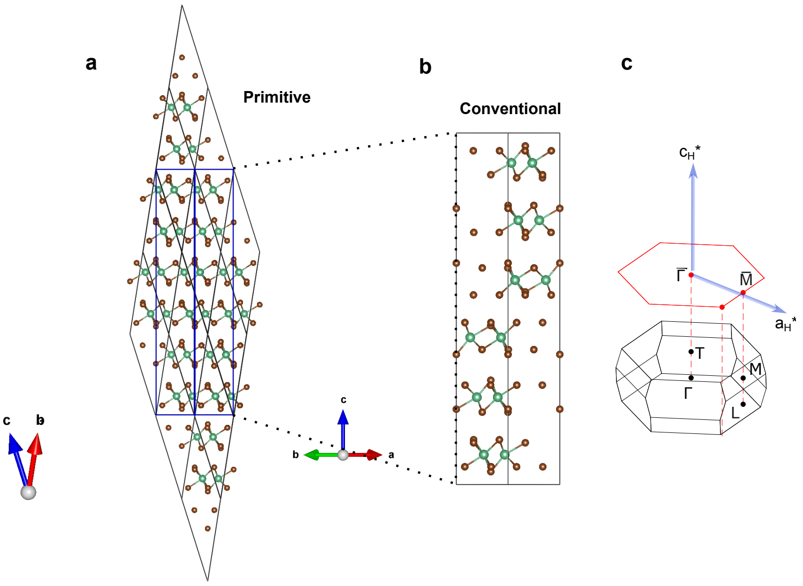

FIG. S1: (a) Primitive supercell of Nb_3Br_8, where each rhombohedral unit represents the unit cell. The six-layered conventional unit cell (b) can be constructed from the primitive one. (c) Brillouin zone of the rhombohedral unit cell with its hexagonal projection (red hexagon) following the interconversion between the two representations of the trigonal crystal system.

where,

$$O = \begin{pmatrix} 1 & -1 & 0 \\ 0 & 1 & -1 \\ 1 & 1 & 1 \end{pmatrix} \tag{S1c}$$

The unit cell obtained upon transforming to the hexagonal coordinates is effectively a supercell consisting of three units of the primitive rhombohedral unit cell. The relationship between these representations can be shown pictorially for Nb_3Br_8, where the six-layered conventional unit cell (see Fig.S1(b)), written in hexagonal coordinates, is constructed from a supercell of the two-layered primitive unit cell, written in rhombohedral coordinates (Fig.S1(a). In connection with the ARPES experiment, we use the conventional unit cell as it offers the convenience of defining the surface normal along the hexagonal c^*_H axis in the reciprocal space, which is parallel to k_z. Besides, the relevant high-symmetry points in different k_z planes in the rhombohedral Brillouin zone can be projected on the in-plane hexagonal reciprocal lattice vector a^*_H (see Fig.S1(c)). Therefore, using the above transformation and defining the projected Brillouin zone allows us to present band dispersion of Nb_3Br_8 (or any rhombohedral system) in the relatively familiar hexagonal setting, simplifying the analysis.

Supplementary Note 2

Here we discuss the model we used to fit the data in Fig.2(i,j) of the main text. In general, the temperature, instrumental resolution and finite photoelectron escape depth play a key role in broadening the momentum and energy dependent parts of the photoemission intensity. While a quantum mechanical treatment of this problem is rather involved, the broadening events mentioned above can be captured tacitly in a simple phenomenological model, as shown below.

$$I(k_i, \varepsilon) = \int dk_z \delta(\varepsilon(k_z) - \varepsilon)\Gamma(k_z, k_i) \tag{S2}$$

For instance. the $\delta(\varepsilon(k_z) - \varepsilon)$ is replaced by a Gaussian with a small smearing representing finite temperature and instrumental energy broadening in the cosinusoidal dispersion $\varepsilon(k_z)$. On the other hand, $\Gamma(k_z, k_i)$ is a Lorentzian describing k_z broadening [S1] in the $k_z = k_i$ plane. We chose energy dispersion to be periodic with 0.44Å^{-1}, and assumed an inelastic mean free path (IMFP) of 5Å for which, the k_z broadening is expected to be approximately

0.2Å^{-1}. The fitted value of the k$_z$-broadening was ≈ 0.35 Å^{-1}, which is reasonably close to our assumed k$_z$ broadening. Thus, the EDCs presented in Fig.2(i,j) of the main text would be consistent with a band insulator with cosinusoidal dispersion along the k_z axis, with a shoulder that develops due to k_z broadening, or alternatively with a Mott-insulator, where the shoulder in the EDC develops due to the splitting of the Hubbard bands by the layer dimerization. Only when considering the analysis of the out-of-plane dispersion presented in Fig. 3, one can clearly distinguish the two cases and conclude that the latter is the correct interpretation.

Supplementary Note 3

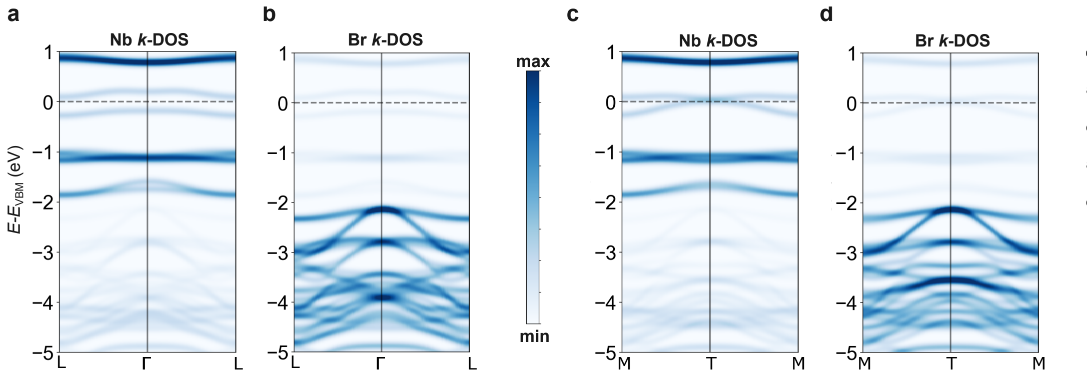

FIG. S2: Contribution of each atomic species in Nb_3Br_8 in the (a,b)Γ-plane, and (c,d) T-plane. k-DOS stands for momentum resolved density of states

To understand the composition of the valence bandstructure of Nb_3Br_8, we can project the partial density of states of individual atomic species by computing the momentum resolved partial density of states of each atomic species, as shown below.

$$A_n^\alpha(k,\varepsilon) = \sum_\nu |\langle\phi_\nu^\alpha(k)|\psi_n(k)\rangle|^2 \delta(\varepsilon - \varepsilon_{nk}) \tag{S3}$$

Here, the Kohn-Sham eighenstate of the nth band $|\psi_n\rangle$ is projected onto the orthogonalized atomic orbital $|\phi_\nu\rangle$ of species $\alpha \in \{Nb, Br\}$, and ε_{nk} are the Kohn-Sham eigenvalues. The summation is performed over all the valence orbitals of species α to obtain atom projected and momentum resolved bandstructures shown in Fig.S2. We can clearly see that the valence bandstructure in the $\Gamma-$ and $T-$planes is governed by the electronic states of Nb. This provides reasonable motivation to restrict the toy model described in the main text to a single-band and single-orbital model.

Supplementary Note 4

In Fig.S3(a), we have shown the k-DOS of Nb atoms projected on the bandstructure of Nb_3Br_8 along the T-Γ-T path, calculated using DFT. However, in our experiment we found that the top of the highest occupied band has a spacing that is twice as large as expected from the DFT calculations. Considering the possibility of electronic correlations, we performed DMFT calculations. We determined the Mott phase by observing the divergence in the imaginary part of the self-energy, as shown in Fig.S3(b). When plotted in the Brillouin zone of the undimerized chain, that is, along the $T^* - \Gamma - T^*$ direction, we show that the spectral function appears at T^* (marked by red arrows in Fig.S3(c)), which makes the apparent band periodicity two times of what we expect (as discussed in the main text). Here, T^* is at the boundary of the Brillouin zone of a hypothetical undimerized material with a lattice constant equivalent to the layer spacing d.

Supplementary Note 5

We present the soft X-ray data of the k$_z$ dispersion in Fig.S4, measured using circularly polarized light. The observed periodicity of the highest occupied band is approximately the same as that observed using VUV radiation

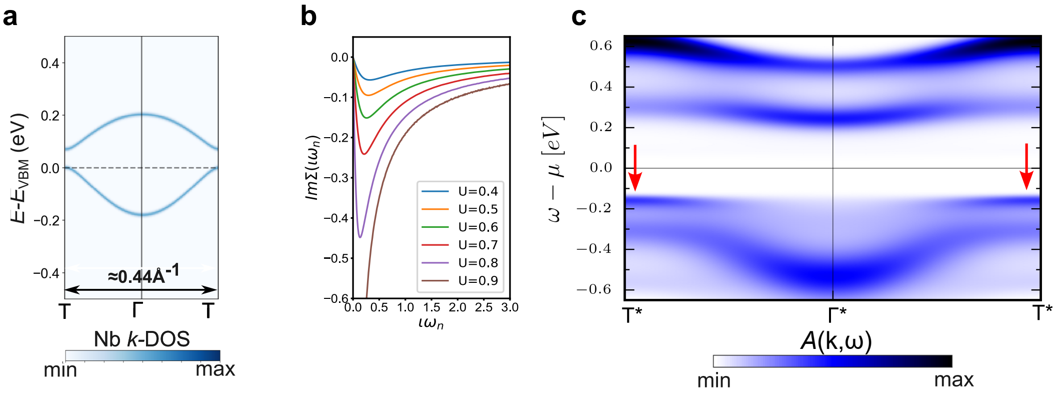

FIG. S3: (a) DFT bandstructure of Nb_3Br_8 along the k_z direction with projected k-DOS od Nb atoms. (b) Evolution of the imaginary part of self-energy with the Hubbard interaction U. (c) DMFT spectral function computed for the dimerized Mott-insulating phase. The red arrows show spectral function appearing at the Brillouin zone edge of the undimerized unit cell.

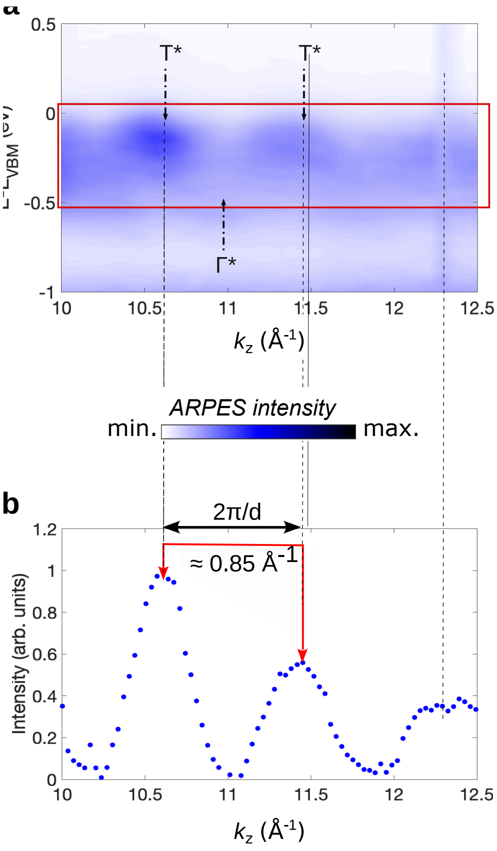

FIG. S4: Soft X-ray ARPES data. (a) The k_z dispersion of Nb_3Br_8 measured using soft X-ray radiation, and the corresponding MDC is shown in (b).

(Fig.3(a) of the main text). We point out that the qualitative differences in the spectral weight between the VUV and soft X-ray data is probably due to the different polarization of the incident light.

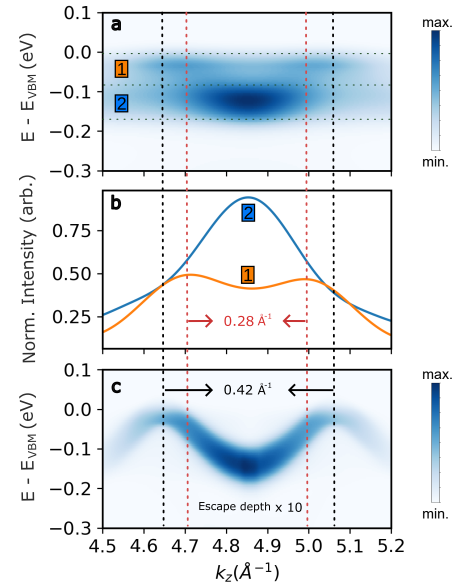

FIG. S5: **Simulated ARPES intensity for band insulating phase a** Simulated intensity with escape depth from universal curve. **b** Corresponding MDC. Two MDC curves use different energy integration ranges as indicated in **a**. **c** Simulated ARPES spectrum with 10 times longer escape depth.

Supplementary Note 6

In the simulated MDC of band insulating phase (Fig.3h of the main text), the predicted periodicity of the top of the valence band is slightly smaller than the naively expected periodicity of π/d (reproduced here in Fig.S5(a)). The reason for that is the intensity modulation of the band due to the band hybridization. This effect can be more clearly observed when reducing the effect of k_z broadening when inspecting the ARPES intensity with a ten times extended escape depth (Fig.S5(c)). From this plot it is clear that the top of the valence band has a spacing of π/d, but the intensity modulation of the band leads to a reduced spacing of the peaks in the MDC.

Supplementary Note 7

The Eq.(7) in the Methods section relates the spectral function $A_{jj'}$ in orbital basis with ARPES intensity. Here we illustrate how the spectral function leads to the observed periodicity in Fig.3 of the main text. We decompose Eq.(7) in the Methods section into the diagonal contribution (jj')=(11),(22) and the off-diagonal (jj')=(12),(21) contribution. In Fig. S6, we again compute ARPES intensity as a funnction of k_z for the band insulator case, and further compare both contribution and the total signal in the limit of infinite escape depth. The off-diagonal terms have the phase modulation $e^{i\mathbf{p}\cdot(\mathbf{r}_j-\mathbf{r}'_j)}$, leading to oscillating intensity as a function of k_z in Fig. S6**a**. On the other hand, such phase modulation vanish for the diagnoal terms in Fig. S6**b** , which is directly proportional to the spectral function $\sum_{j=1,2} A_{jj}$. As a result, the total signal in Fig. S6**c** shows intensity cancelation in some k_z ranges, and produce the π/d period shown in Fig.3**d** and **h**. A similar phase modulation in the off-diagonal terms occurs in the case of dimerized Mott-insulator, and leads to the observed ARPES intensity with period $2\pi/d$. We transfromed the orbital based spectral function (j=1,2) from spectral function in the basis of bonding (b) and anti-bonding (a) states. They are related to each other by $A_{j=j'} = \frac{1}{2}(A_{aa} + A_{bb})$ for diagonal terms and $A_{j\neq j'} = \frac{1}{2}(A_{bb} - A_{aa})$ for off-diagonal terms.

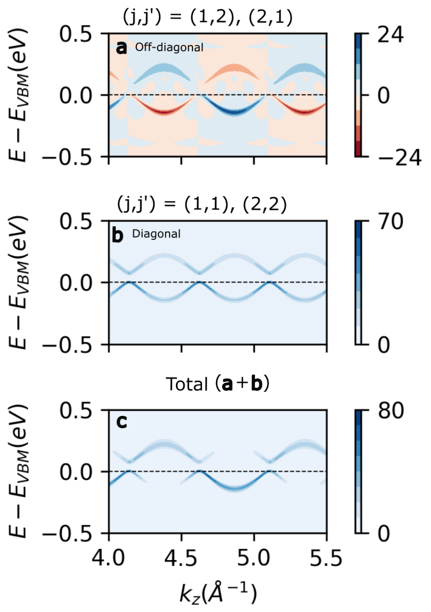

FIG. S6: **Diagonal, off-diagonal contributions, and the total ARPES intensity for the band insulator.** **a** The diagonal terms (jj')=(11),(22) in ARPES intensity. **b** The off-diagonal terms (jj')=(12),(21) in ARPES intensity. **c** Total ARPES intensity. Here the intensity is evaulated with infinite escape depth.

[S1] V. Strocov, Journal of Electron Spectroscopy and Related Phenomena **130**, 65 (2003), ISSN 0368-2048, URL https://www.sciencedirect.com/science/article/pii/S0368204803000549.
